# HPV Prevention Strategies in 2024: An Approach by the University of Milan

**DOI:** 10.3390/vaccines14040362

**Published:** 2026-04-18

**Authors:** Pier Mario Perrone, Ilaria Casolaro, Serena Pescuma, Ilaria Bruno, Martina Cappellina, Enrico Lupo Maria Caprara, Giovanni Cicconi, Andrea Cinnirella, Alessandro De Monte, Francesca Maria Grosso, Elvira Pantó, Andrea Pedot, Enrico Pigozzi, Simona Scarioni, Sudwaric Sharma, Catia Rosanna Borriello, Fabrizio Pregliasco, Silvana Castaldi

**Affiliations:** 1 Department of Clinical Sciences and Community Health, University of Milan, 20122 Milan, Italy; 2 Department of Pathophysiology and Transplantation, University of Milan, 20122 Milan, Italy; 3 Department of Biomedical Sciences for Health, University of Milan, 20122 Milan, Italy; 4 Vaccination Unit, ASST Fatebenefratelli Sacco, 20131 Milan, Italy; 5 Welfare General Directorate, Lombardy Region, 20100 Milan, Italy; 6 Quality Unit, Foundation IRCCS Ca’ Granda Ospedale Maggiore Policlinico, 20122 Milan, Italy

**Keywords:** human papillomavirus, vaccination campaign, university, Italy, catch-up strategy

## Abstract

**Background/Objectives**: Human papillomavirus (HPV) infection is a major concern in public health, given its role as a persistent sexually transmitted infection and a causative agent of non-cancerous and cancerous lesions (neoplasms). The increasing infection rates observed in recent years underscore the need for effective public health measures to address this issue. The objective of this study is to describe the challenges and the results of conducting vaccination campaigns within a university setting and its impact on the HPV vaccination rate. **Methods**: A multifaceted approach was adopted, entailing the implementation of two distinct interventions. Following the promotional and educational online campaign (described elsewhere), vaccination delivery took place from November 2024 to July 2025 in the university campus and in three university hospitals in Milan. Overall and covariate-specific drop-out rate is calculated; significance is tested through a chi-square test of homogeneity between the population that completed less than three doses vs. those who completed the full cycle. Overall and vaccine-specific vaccination proportion is reported. Results: The vaccination rate for first doses reached 92% of available appointments, with a slight female majority (50.9%) and the 23–26 age as the most represented group (47%). The most represented nationality was Italian (58.4%), followed by Iranian (26.5%). Regarding the vaccination sites, the university venue recorded the highest rates in terms of both vaccines booked (56.4%) and vaccines administered (64.7%). With a net loss in follow up, consistent with WHO data, the three-dose HPV vaccination campaign was completed by 82.5% of participants. A chi-squared test of homogeneity revealed significant differences in age distribution between vaccination groups, χ^2^ (3) = 347.78, *p* < 0.001, Cramér’s V = 0.457. Participants who received only one dose were predominantly younger (17–22 years: 71.1% vs. 19.0%, difference = 52.1 percentage points, 95% CI [46.6, 57.7]). Meanwhile, a catch-up strategy raised interest on other crucial vaccinations. **Conclusions**: The findings pertaining to the vaccination rate underscore the heightened awareness among young adults concerning the HPV vaccine. They further substantiate the efficacy of the integrated strategy encompassing advisory and educational site-based campaigns as an initial measure to attain the WHO-endorsed vaccination rates.

## 1. Introduction

Human papillomavirus (HPV) is a DNA virus that is considered one of the most prevalent sexually transmitted infections (STI), posing a significant challenge to public health [1]. There are more than 200 types of HPV [2], of which 15 are defined as being at oncogenic risk in both sexes [3]. HPV accounts for close to 700,000 cancer cases each year worldwide, including 236,500 new cases and 494 deaths in Italy, representing 1.3% of all cancers in the female gender, with up to 80% of sexually active individuals contracting the infection in their lifetime [4]. In most cases, the infection is transient, asymptomatic and regresses spontaneously within a period of two years; however, there are instances where it persists or progresses [5,6].

As a STI, the main risk factors for HPV infection are linked to sexual behavior, such as starting sexual activity at a young age and having a high number of sexual partners over a lifetime [7,8]. Consequently, approximately 50% of new cases occur among individuals aged 14–25 years [9], constituting a substantial threat to public health from both clinical and economic standpoints. The significant clinical and economic repercussions, in light of the widespread access to vaccines, underscore the efficacy of vaccination as the most effective preventive measure [10,11,12].

In Italy, the Piano Nazionale Prevenzione Vaccinale (PNPV 2023–2025) [13] has been developed in accordance with the World Health Organization’s (WHO) guidelines for the control of HPV infection, with the objective of ensuring that all adolescents receive the recommended HPV vaccination by the age of 15 [4]. Despite the pivotal role of the vaccine in these demographics, particularly within the sexually active population, the Italian context is characterized by a conspicuously low vaccination coverage rate, especially among young adult males. This finding is consistent with international data.

In order to address this issue, the WHO has supported the implementation of initiatives aimed at primary prevention, including facilitating access to the HPV vaccine through the organization of open-access vaccination days, with particular attention to populations considered at risk of infection [12,14,15,16].

Data updated to 31 December 2024 about vaccination coverage against HPV in Italy referring to birth cohorts from 2000 to 2012 show vaccination rates. The scientific literature supports this kind of initiative by reporting examples of studies relating to vaccination proposals against HPV aimed at university students [14]. These initiatives are based on the idea of vaccinating small samples from specific categories, encouraging them to become vaccine advocates among people they meet.

Vaccination in school and university settings has emerged as a strategic and effective approach to reaching adolescents and young adult populations that are often difficult to engage through traditional healthcare channels [17,18,19]. Experiences from different countries demonstrate how educational institutions can serve as critical platforms for both emergency responses and catch-up immunization strategies: USA, France and Italy teaching-site vaccination programs resulted as valuable tools for delivering and reinforcing immunization strategies. In particular, in addition to improving access to HPV vaccination for a key demographic, campus-based initiatives enable targeted educational and outreach activities, fostering greater awareness and informed decision-making precisely during the years when young people begin to make independent decisions about their own health [20,21]. It is imperative to underscore the potential of such programs to mitigate disparities in the access to vaccines among students confronted with financial constraints, temporal limitations, or a paucity of awareness regarding the available services [22].

The aim of this study is to describe the implementation of a vaccination campaign against HPV, targeting students attending the University of Milan (Italy), and its results trying to assess the reasons for its success and the possible intervention to increase adherence in future campaigns.

## 2. Materials and Methods

The vaccination campaign was implemented from 18 November 2024 to 21 July 2025. Alongside it, a descriptive observational study was conducted to collect relevant covariates to better understand its impact. A combination of two different vaccination strategies was adopted: ad hoc outpatient ambulatory at the university and on-site ambulatory at local hospitals, open from 9 AM to 5 PM on weekdays. A communication campaign was launched in October 2024 via the university website, social media and QR-coded panels on-site to inform students of the times and locations. Access to the vaccination centers was by appointment, bookable via a university application with a link on the university’s information page, and direct.

The administration of the first doses of the HPV vaccine ended on 13 January 2025, due to last-minute adjustments during the campaign related to contingencies (vaccine supply problems) that led to changes in the planned calendar. Three vaccination teams, made up of two public health residents for each workstation to collect the medical history and administer the vaccination, worked in different outpatient clinics, reaching a total of 30 subjects per hour who were vaccinated, with a 9-valent vaccine and with a maximum of 210 bookable slots per day, managed by an Excel agenda. The supply of vaccines was guaranteed by the local reference hospital center and delivered daily to the designated vaccination site, with particular attention to respecting the cold chain.

A written informed consent form for each vaccination was obtained and medical history was collected. Vaccinations were recorded in the digital regional platform SIAVR 4.45.4.0 (until end of 2024, when it was migrated to a more advanced one), and on Arvax (from 2025, which is currently in use after the software update). Vaccination data were collected from the first day of the campaign to verify the accuracy of the reported numbers compared with the vaccinations actually administered. Aggregated data on age (i.e., 17–22, 23–26, 27–30, 31+), sex and nationality were obtained from the data extracted from the software. Percentages of booked vaccinations, unbooked vaccinations and no-shows were also calculated; immediate adverse reactions were taken into account limited to the first doses.

Second HPV doses were administered in January-March 2025 two months after the first one, and the third HPV vaccinations were performed in May–July 2025 four months after the second doses. The vaccination campaign, regarding the following HPV doses, was carried out in vaccination centers near the university campus and in the same ad hoc ambulatories in the hospitals where the first doses were provided. In these occasions, participants were involved in a catch-up strategy for other crucial vaccinations, after checking their vaccination certificates.

Baseline aggregate data about the university students’ population were obtained in anonymized file through the university office. Individual-level general demographic data (gender, age group, nationality) were obtained for each person that received at least one dose of the HPV vaccine, alongside the vaccination site and which vaccines were administered.

All statistical analyses were performed using R software, version 4.3.2. Categorical variables were summarized as raw numbers and percentages. Descriptive tables are presented for the overall Milan university student population and for the cohort of students that were vaccinated with at least 1 dose.

Overall and category-specific dropout ratio was then calculated. To highlight significant differences in covariates associated with completing the full cycle vs. receiving less than 3 doses, contingency tables are shown. A chi-squared test of homogeneity was then calculated (with a *p*-value of 0.05 to reject the hypothesis that the categorical variables are distributed in the same way across the two populations. Cramer’s V was calculated as a measure of effect size for the association between vaccination status and each demographic variable. To identify which specific categories contributed to significant overall differences, 95% confidence intervals were calculated for the difference in proportions between groups using the Wald method. The estimates are not adjusted for multiple comparisons.

The proportion of students that received catch-up vaccination alongside the HPV doses is reported. Vaccine-specific proportions are shown in a separate figure.

Ethical approval was not required for this study under Italian law [23,24].

## 3. Results

The student cohort of the University of Milan in 2024 was distinguished by a higher proportion of male students and a higher prevalence of individuals under the age of 27. Among these, 1805 students booked an appointment for their first HPV vaccination through the university website following the promotion of the HPV vaccination campaign 2024 on the University of Milan’s official media channels.

Baseline characteristics are reported in Table 1 and Table 2 as raw numbers and percentages (*p* < 0.05). Of 1667 students, 818 were males and 849 were females. The most represented age group was 23–26 years (47%).

**Table 1:** Baseline description of the university student population.

	Milan University Student (N = 66,357)
Sex	
M	38,566 (58.1%)
F	27,791 (41.9%)
Age	
17–22	31,831 (48.0%)
23–26	19.694 (29.7%)
27–30	7787 (11.7%)
31+	7045 (10.6%)

**Table 2:** Baseline description of the subjects immunized in the vaccination campaign against HPV, carried out in the University of Milan, Italy (n = 1667).

	Subject Vaccinated with at Least 1 Dose (N = 1667)
Sex	
M	818 (49.1%)
F	849 (50.9%)
Age	
17–22	479 (28.3%)
23–26	783 (47.0%)
27–30	263 (15.8%)
31+	149 (8.9%)

**Figure 1:**
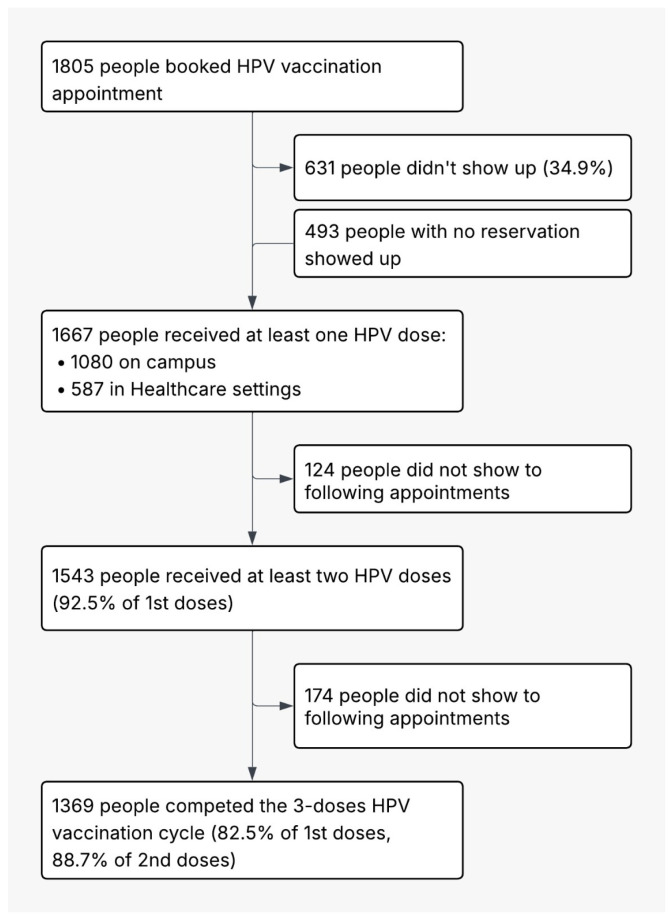
Campaign flowchart of the vaccination cycle.

At the conclusion of the vaccination program, 92.5% of recipients had received two doses, while 82.5% of students who received the first dose and 88.7% of students who received the second dose had completed the entire vaccination cycle, as illustrated in Figure 1. At the same time, the observed rate of vaccine dropout was 7.5% between the first and second doses, and 11.7% between the second and third doses.

Chi-squared tests of homogeneity revealed significant differences between the one-dose and three-dose groups across all demographic variables examined (Table 3). The distribution of sex differed significantly between groups, χ^2^ = 5.74, *p* = 0.017, though the effect size was negligible (Cramér’s V = 0.059). The uncompleted cycle group had a higher proportion of females (57.4%) compared to the three-dose group (49.5%), with a difference of 7.9 percentage points (95% CI: 1.6% to 14.1%). Nationality distribution also differed significantly between groups, χ^2^ (2) = 18.72, *p* < 0.001, with a small effect size (Cramér’s V = 0.106). The most pronounced difference was observed for age distribution, χ^2^ (3) = 347.78, *p* < 0.001, with a greater effect size (Cramér’s V = 0.457). Participants aged 17–22 years were substantially overrepresented in the uncompleted cycle group (71.1% vs. 19.0%; difference: 52.1 percentage points, 95% CI: 46.6% to 57.7%), whereas those aged 23–26 years were markedly underrepresented (10.1% vs. 55.0%; difference: −44.9 percentage points, 95% CI: −49.3% to −40.6%). The 27–30 age group was also underrepresented in the uncompleted-cycle group (10.1% vs. 17.0%; difference: −7.0 percentage points, 95% CI: −10.9% to −3.0%), while no significant difference was found for participants aged 31 years and older (8.7% vs. 9.0%; difference: −0.3 percentage points, 95% CI: −3.8% to 3.3%).

**Figure 2:**
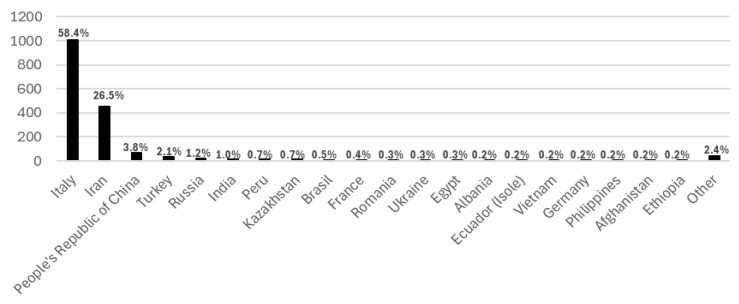
Nationality percentages among participants in the vaccination campaign.

Italian students were the most represented nationality, accounting for 58.4% of the participants, followed by a well-represented Iranian student population, corresponding to merely 26.5% of the student body with other nationalities contributing lower percentages, as detailed in Figure 2 below.

**Figure 3:**
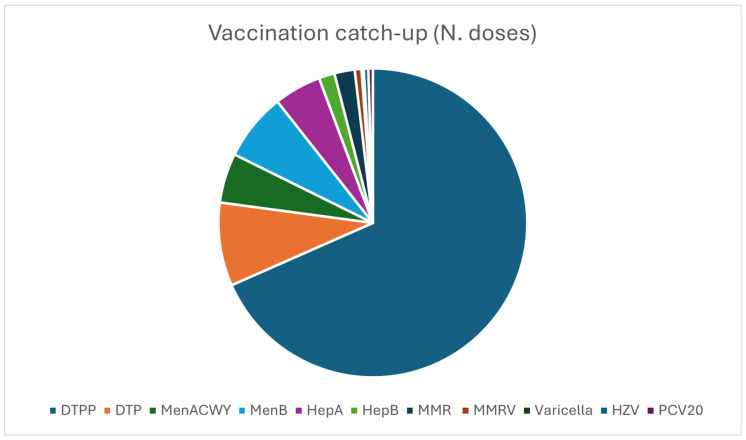
Vaccination catch-up doses: DTPP 290, DTP 37, MenACWY 22, MenB 30, HepA: 21, HepB 7, MMR 9, MMRV 3, VARICELLA 1, HZV 2, PCV20 2.

A total of 4579 HPV doses were administered. Furthermore, other vaccinations were proposed as a catch-up strategy during the second and third HPV doses sessions (see Figure 3).

## 4. Discussion

HPV is considered one of the most significant health issues on a global scale, primarily due to its high prevalence and its role in linking infections to the development of new lifestyle diseases, including cancer, in young populations. The existence of a safe and effective vaccine means that it is possible to reduce the burden of HPV infection and related cancer through the implementation of extensive vaccination campaigns among high-risk populations.

In order to comprehend the viability and efficacy of such interventions, it is imperative to consider the findings of studies undertaken among university students. In this regard, the scientific literature provides numerous examples of vaccination campaigns conducted in educational institutions, proposing on-site models against various infectious agents [25]. These examples demonstrate the efficacy of the proposed approach in terms of adherence. The implementation of seasonal influenza vaccination programs in educational institutions in the USA has been documented [26,27]. Furthermore, in Italy during the COVID-19 pandemic, particular emphasis on influenza vaccination initiatives arose [28,29], in addition to the administration of the COVID-19 vaccines [30,31,32], within educational hospitals. These efforts have encompassed the education of students and medical residents, who have been instructed by healthcare professionals.

In the USA, on-campus vaccination campaigns against meningococcus, primarily type B [33,34], were implemented following infection outbreaks on university premises [35,36]. Conversely, in France, opt-in vaccination campaigns against meningococcus (type W) were organized on university campuses [37] and at middle schools [38]. As evidenced by the implementation of a school-based campaign against HBV in the USA [39], a concerted effort has been made to address this issue on a national scale. A measles and rubella catch-up immunization campaign was conducted in UK universities [40]. Furthermore, a similar campaign was implemented in Swiss universities, which included the addition of mumps to on-campus immunization programs.

The results of our study demonstrate that universities, and educational and workplaces in general, represent an increasingly attractive setting for administering the HPV vaccine to adolescent populations. This is due to both their logistical convenience and the peer emulation effects that a young adult setting can have on those populations who are doubtful or not entirely convinced. The presence of role models who facilitate the expression of doubts and opinions, while also serving as conduits for information or advertising campaigns, enables the navigation of socio-cultural contexts, thereby overcoming any doubts or prejudices that may arise from them. However, it is important to note that in many countries, experience in this regard is lacking, thus highlighting the importance of the present work.

Despite the relatively limited access to the vaccination campaign (2.51%) among the substantial student population at the University of Milan (Italy) and the constrained availability of classrooms for the vaccination campaign, which were only guaranteed for a few days, the percentage of individuals who participated in the three-dose vaccination program exceeded 80%. This result is indicative of a heightened level of interest in HPV vaccination among young adults following an adequate education intervention on the topic. This observation underscores the efficacy of promotional initiatives in raising awareness on vaccination programs.

With regard to demographic characteristics, a slight predominance of female students was observed, though this difference had negligible practical significance. This finding is nonetheless consistent with the literature in which female students appear to demonstrate a propensity to seek preventive healthcare services, consequently diminishing their likelihood of receiving the HPV vaccine [16]. Furthermore, the perception of HPV as a virus that mainly infects females is evidenced by the high prevalence of cervical cancer caused by HPV and poor health literacy on the subject. This perception appears to explain the greater attention paid to vaccination in females.

The most striking finding concerned age distribution. Younger participants (17–22 years) were overrepresented among those who received only one dose, whereas those aged 23–26 years were significantly more likely to have completed the cycle. This pattern may be explained by the introduction of free vaccinations until the age of 26 in the Lombardy region, which likely reaches previously unvaccinated individuals who may have been influenced by family or cultural biases. The lower completion rate among younger adults may reflect a tendency to delay healthcare decisions.

Furthermore, the present study demonstrates the varying degrees of attention accorded to HPV vaccination among students of differing nationalities. Italian students were more likely to complete the cycle, while students of other nationalities showed lower completion rates. This phenomenon may be attributable to a confluence of socio-cultural and healthcare organization factors. The potential for receiving a vaccine free of charge, as opposed to the scenario where it would be administered at a fee in their country of origin, along with the existence of a vaccination campaign, may offer a rationale for the heightened focus on vaccination observed in the first doses among the foreign population. A notably elevated participation rate among Iranian students compared to their proportion over the whole University of Milan population was observed. A significant proportion of these students expressed a strong inclination to receive the vaccination, and they constituted the higher proportion of participants who attended without prior reservation. Notably, Iranian students showed comparable completion rates to the overall sample, possibly due to stronger community networks as the largest foreign student group at the University of Milan. This result is consistent with the findings of previous studies, which have indicated a high level of sensitivity in both sexes within this nationality population with regard to the HPV-related burden [41].

With a net loss in follow up, consistent with WHO data [42], the three-dose HPV vaccination campaign was completed by the large majority of participants. Meanwhile, our experience has shown a successful catch-up strategy implementation. Starting from the analysis of participants’ vaccination certificates in the occasion of second and third HPV doses implementation, other crucial vaccinations have been proposed and administered, thus improving vaccination coverage against infections recognized worldwide as epidemic-outbreak risk factors in community environments [43]. As regards other subjects with no certificate available, through medical history we identified students missing some vaccinations (e.g., not having received the dTpa (Diphteria, Tetanus and acellular Pertussis) one for more than 10 years). Students showed great interest in being adequately vaccinated and no one refused the proposal for other vaccinations. This finding lends further credence to the efficacy of the so-called catch-up strategy, which posits that interactions with patients, irrespective of whether they pertain to health or non-health matters, such as document renewals, serve as opportunities for health education and for addressing vaccinations that have not yet been administered. This assertion is further substantiated by a substantial body of scientific research [44,45].

This was a pilot study aimed at testing the implementation model in the university setting with a catch-up strategy to protect from HPV those who, for various reasons, did not take part in it yet. HPV vaccination is already part of National and Regional Vaccination Prevention Plans, and other targeted regional initiatives. Thus, even if the university does not have routine access to individual vaccination status of its students, vaccinating the 2.5% of enrolled students represent a net increase on the existing coverage.

Notwithstanding the findings and their significance, this investigation is encumbered by certain limitations. These arise from the monocentric nature of the study, which precludes the ability to provide a comprehensive representation of the national or northern Italian scenario. Additionally, the restricted number of available doses is a confounding factor, as it limits the scope and generalizability of the study’s results. Losses to follow up could partly be explained by the fact that the second and third doses were administered at a different site, with respect to where the previous doses were provided. Furthermore, the differences between the first, second, and third doses are attributable to students who may have already received the first and/or second dose at their vaccination center (or on other occasions) and only adhered in our campaign for the missing doses, in order to complete the vaccination cycle. Lastly, gender categories only describe binary identities, with no further investigation on non-binary ones.

## 5. Conclusions

HPV is a significant public health concern due to its high prevalence and associated complications. The existence of a safe and effective vaccine is a prerequisite for the prevention of infection, a goal which can be realized through the attainment of a high vaccination coverage rate, particularly among young adults. WHO recommends a single dose of the HPV vaccine for people not at risk as an effective alternative to the two-dose series: *“Alternative single-dose schedule. As an off-label option, a single-dose schedule can be used in girls and boys aged 9–20 years. Current evidence suggests that a single dose has comparable efficacy and duration of protection as a 2-dose schedule and may offer programme advantages, be more efficient and affordable, and contribute to improved coverage. From a public health perspective, the use of a single dose schedule can offer substantial benefits that outweigh the potential risk of a lower level of protection if efficacy wanes over time, although there is no current evidence of this”* [41].

The teaching site-based vaccination program has been demonstrated in the literature to represent a potential resource for the implementation of catch-up strategies for several important vaccines. This study demonstrates the significance of university-based vaccination campaigns in reaching a pivotal demographic for the HPV vaccine. It also underscores the importance of educational and communication initiatives in promoting awareness and understanding. It has also represented the base to assess whether the university setting could be a useful tool for proceeding with the catch-up relating to crucial vaccinations in a population of subjects who, due to economic reasons, lack of time or awareness, vaccination access may be limited and not equal.

This pilot study, regarding only 2.5% of the total university population, could represent the first step towards the implementation of an annual HPV university vaccination campaign in Italy, with the objective of increasing attention from policymakers regarding overall vaccination coverage among young adult students, as a potential hard-to-reach population.

## Figures and Tables

**Table 3:** Chi-squared test results and effect size (Cramér’s V) of the association between baseline variables and probability of completing the vaccination cycle.

	Subjects Vaccinated with Less than 3 Dose (N = 298)	Subjects Vaccinated with 3 Doses (N = 1369)	Χ^2^ (Degrees of Freedom)	*p*-Value	Cramérs’s V
Sex					
M	127 (42.6%)	691 (50.5%)	5.74 (1)	0.017	0.059
F	171 (57.4%)	678 (49.5%)			
Nationality					
Italy	147 (49.3%)	809 (59.1%)	18.72 (2)	<0.001	0.106
Iran	82 (27.6%)	374 (27.3%)			
Other	69 (23.1%)	186 (13.6%)			
Age					
17–22	212 (71.1%)	260 (19.0%)	347.78 (3)	<0.001	0.457
23–26	30 (10.1%)	753 (55.0%)			
27–30	30 (10.1%)	233 (17.0%)			
31+	26 (8.7%)	123 (9.0%)			

## Data Availability

The raw data supporting the conclusions of this article will be made available by the authors upon request.

## Abbreviations

The following abbreviations are used in this manuscript:

HPV	Human Papillomavirus
STD	Sexually transmitted diseases
